# UNDERSTANDING HEALTHCARE UTILIZATION, FRAILTY, AND DELAYS AMONG OLDER SEXUAL AND GENDER MINORITY ADULTS

**DOI:** 10.1093/geroni/igad104.3760

**Published:** 2023-12-21

**Authors:** Chelsea Wong, Louisa Smith, Robert Cavanaugh, Brianne Olivieri-Mui

**Affiliations:** HebrewSenior Life, Boston, Massachusetts, United States; Northeastern University, Boston, Maine, United States; Northeastern University, Boston, Massachusetts, United States; Northeastern University, Boston, Massachusetts, United States

## Abstract

Older sexual and gender minority adults (OSGM) have a higher burden of frailty, mental health conditions, and healthcare delays compared to non-SGM older adults (non-OSGM). The study aimed to evaluate the impact of health disparities related to frailty and delays on the relationship between OSGM and healthcare utilization, we hypothesized that improving frailty and delays would reduce healthcare utilization. We used marginal structural models in the All of Us version 6 Controlled Tier Dataset to study the mediating role of frailty and delays on healthcare utilization (self-reported general doctor (PCP) and mental health (MH) visits) by SGM status. OSGM (n=4,763) compared to non-OSGM (n=68,146) were younger (mean [SD], 63 [8] vs 66 [8]), had higher frailty (26% vs 19%), reported more delays (30% vs 24%), less excellent mental health (23.2% vs 30.2%), and majority were insured (96% vs 97%). Minimal differences between the total effect and controlled direct effect between SGM status and visits for frailty (PCP -0.01, MH 0.15) and delays (PCP 0, MH 0.16), suggests visits would remain similar if all OSGM were not frail or reported no delays. Contrary to our hypothesis, improving frailty and delays did not impact healthcare utilization among OSGM. Factors other than frailty and healthcare delays may be influencing healthcare utilization among OSGM in All of Us. Further, bias due to self-report/volunteer bias may affect the ability to examine the association of interest. Future work should examine the consensus between survey and EHR reported healthcare utilization within All of Us.

